# Investigating the effects of ultrasound and alkaline pH on protein recovery from *Acipenser baerii* heads: Insight into ultrastructural, functional and antioxidant attributes

**DOI:** 10.1016/j.ultsonch.2025.107479

**Published:** 2025-07-25

**Authors:** Seyedeh Mona Hosseini Choupani, Masoud Rezaei, Samaneh Pezeshk, Shahab Naghdi, Reza Tahergorabi

**Affiliations:** a Department of Seafood Processing, Faculty of Marine Sciences, Tarbiat Modares University, P.O. Box 46414-356, Noor, Iran; b Food and Nutritional Sciences Program, North Carolina Agricultural and Technical State University, Greensboro, NC, USA

**Keywords:** pH shift, Ultrasound, Protein isolated, Functional attributes, Antioxidant properties

## Abstract

This study investigated the effects of ultrasound combination and alkaline pH adjustment on protein extraction from Siberian sturgeon (*Acipenser baerii*). Optimal protein extraction, solubility, and essential amino acid content were achieved at pH 11.5, within the tested range of 10.5 to 12.5. Ultrasound, applied at power levels ranging from 100 to 400 W, enhanced protein yield and functional properties, including foaming, which peaked at 65.35 % at pH 11.5, and emulsification, which increased to 82.50 % when combined with 400 W ultrasound at the same pH. Additionally, gelation properties and water-holding capacity were improved. The treated proteins also exhibited significantly enhanced sensory quality compared to the control group. Increased ultrasound power correlated with reduced particle size, altered protein structure, and higher protein recovery. Moreover, 400 W ultrasound significantly boosted antioxidant properties, as observed through ABTS and DPPH free radical assays at pH 11.5, indicating a correlation with inhibition percentage, as evidenced by increased radical scavenging capacity and reduced iron levels. Consequently, the combined application of alkaline pH adjustment and ultrasound processing significantly improved the sensory, functional, and antioxidant properties of proteins extracted from Siberian sturgeon.

## Highlights

• Ultrasound enhances *Acipenser baerii* protein extraction at pH 11.5.
• Optimal protein solubility/amino acids achieved at pH 11.5.
• 400 W ultrasound boosts protein antioxidant properties.
• Ultrasound improves protein foaming and emulsification.
• Combined pH/ultrasound enhances sensory quality of proteins.

## Graphical abstract

**Figure:**
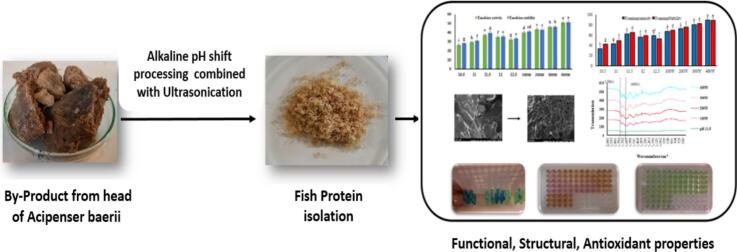

## 1 Introduction

The growing global population has led to an increased demand for food sourced from both marine and freshwater environments. Industrial seafood processing is considered one of the most important and economically significant commercial industries in the world. Regrettably, a substantial quantity of both wild and farmed fish, estimated at 22.2 million tons, is discarded as non-edible waste [1]. The Siberian sturgeon (*Acipenser baeri*), a cold-water fish, is highly valued and commands significant economic importance in many countries worldwide [2]. Its ability to withstand environmental fluctuations and its good growth potential make it a popular aquaculture species. Additionally, the fish and crustacean processing industry produces up to 70 % in by-products, including heads, skin, fins, scales, viscera, and shells, which are often thrown away or repurposed as silage or fish meal [1,3]. This situation poses a significant threat to both the environment and human health while also failing to recognize the potential value of these by-products [3]. These by-products represent a substantial source of valuable bioactive compounds, including proteins, fish oil, carotenoids, carotenoproteins, and polysaccharides such as chitin and chitosan [1,4]. Fish protein contains significant amounts of essential amino acids, highly valued for human consumption; Therefore, focusing on protein extraction from fish processing by-products is considered an ideal approach by industry and researchers alike [5,6]. Various protein products, such as hydrolyzed protein, protein concentrate, and protein isolate, are made from by-products of aquatic sources. These products differ in terms of their protein content and sensory properties [6].

Fish protein isolate is a protein compound extracted from fish materials, often utilized as an additive in various food industry products. This isolated protein has distinctive functional properties that enhance its usefulness in food applications [7]. Common methods for extracting proteins from fish processing by-products include pH shift and isoelectric precipitation. These techniques leverage the solubility of muscle proteins in water at high pH levels (above 10.5) or low pH levels (below 3.5), enabling the separation of non-soluble residues like fat, bone, and skin through centrifugation [8]. Although this process effectively removes bones and fats, the protein yield generally falls between 50 % and 70 %, influenced by factors such as the solubilization pH, the type and quality of the raw materials, and the specific fish species used [9,10].

The ultrasound process is a non-thermal, non-toxic, and safe physical method for modifying proteins, which helps decrease energy consumption and reduce processing time. Cavitation, caused by ultrasound waves, creates bubbles that collapse, generating macro turbulence, rapid particle collisions, and micro-porosity [10]. This effect improves the solvent’s ability to penetrate the cellular matrix and enhances interactions between target compounds, like proteins, and the solvent. By optimizing the conditions for ultrasound application, it’s possible to modify the resulting protein isolate, potentially enhancing its functional properties [10,11].

Since the head of the *A. baerii* fish represents a substantial portion of its weight and is regarded as the primary by-product, the objective of this study was to investigate the effects of ultrasound waves in conjunction with pH shift processes on the yield, structure, functional properties, and antioxidant activity of the isolated proteins derived from it.

## 2 Materials and methods

### 2.1 Materials

The heads of Siberian sturgeon (*A. baerii*) were procured from the Siberian Sturgeon Farm located in Mahmoud Abad, Iran. To ensure their freshness, the by-products were immediately transported to the laboratory in boxes packed with pieces of ice. Upon arrival, the samples were promptly minced using a grinder (CBJR-120, Chuang Bo, China) equipped with a grid that had 5 mm holes. The resulting minced material was mixed and then stored in plastic bags at a temperature of −80 °C.

### 2.2 pH shift processing integration with ultrasound

By-products were processed using a modified pH-shift method described by Pezeshk et al. (2021). Sixty grams of minced material were blended with 360 mL of chilled distilled water (1:6 w/v) for 5 min at 10.000 rpm using a Polytron Homogenizer (IKA, Germany) immersed in an ice bath. The pH was then adjusted to 10.5, 11, 11.5, 12, and 12.5 using 2 M NaOH, followed by an additional 5 min of stirring. Each homogenate was centrifuged at 8500*g* for 20 min at 4 °C (Universal Premium 20000R) to isolate the soluble proteins. The soluble proteins were then adjusted to pH 5.5, centrifuged again under the same manner, freeze-dried (CREST, Switzerland), ground into a powder, and stored at −80 °C. A combination of pH-shift processing and ultrasound treatment was executed with the modifications. Initially, the by-products were combined with cold water in six different ratios, homogenized, and their pH calibrated to 11.5, as previously elucidated [10]. Subsequently, the homogenates were subjected to ultrasound treatment using a HELSCHER ultrasonicate (UP400S, America) at frequencies of 20 kHz and power levels of 100, 200, 300, and 400 W for a total duration of 20 min. The sonication process involved a cyclic pattern of operation, with 2 s of sonication followed by 3 s of pause. To prevent excessive heating, the homogenates were placed in a 400 ml beaker, ensuring that they were just below the ultrasound probe and immersed in a solution at a depth of approximately 2.0 cm. An ice bath was utilized to maintain a controlled temperature. Following sonication, the samples underwent centrifugation, pH adjustment, and moisture removal as previously outlined. Finally, all samples were lyophilized and stored at −80 °C.

### 2.3 Assessment of extraction yield and protein solubilization

The efficiency of protein solubilization and protein recovery was evaluated by quantifying the protein levels in the original mixture following homogenization under the specified pH conditions (H), and in the isolated protein. The quantification was conducted using the Kjeldahl method, following the guidelines established by the AOAC (1990). Furthermore, the protein levels in the first supernatant (S1) and the second supernatant (S2) were determined using the Lowry method [12], with some modifications based on the protocol introduced by Markwell et al. (1978).

### 2.4 Amino acid composition

The amino acid composition of the proteins was determined following the procedure outlined by Surasani (2018).

### 2.5 Foaming properties

The protein samples' foam stability and foam capacity were assessed. Specifically, 1 g of each protein powder was mixed in 10 ml of pure water and thoroughly blended using a T25 Digital homogenizer at a speed of 15,000 rpm for 2 min [14].

### 2.6 Emulsifying properties

The emulsifying activity index (EAI) and emulsifying stability index (ESI) of the isolated proteins were determined with slight modifications to the method described by Rajasekaran et al. [15]. Three mL of corn oil were homogenized with 5 mL of a 1 % (w/v) fish protein solution using a laboratory homogenizer at 15.000 rpm for 2 min at room temperature. Immediately and after 10 min, 50 µL of the emulsion were sampled from the bottom of the container, homogenized in 5 mL of 0.1 % (w/v) sodium dodecyl sulfate (SDS) solution, and vortexed for 10 s. The absorbance of the solution was then measured at 500 nm using a spectrophotometer. EAI and ESI were calculated using following equations, respectively.

$$EA\;\left(\frac{m2}{g}\right)\;=\;\frac{2\times 2.303}{C\;\times (1-\varphi )\times 10}A0\;\times dilution$$

$$ESI\;\left(\%\right)\;=\frac{A10}{A0}\;\times 100\;$$

### 2.7 Gel formation

Following the method described by Benelhadj et al. [16], we determined the minimum fish protein isolate concentration needed to form a gel. Various concentrations of different isolated proteins (2 % to 20 %, w/w) were prepared, heated for an hour, rapidly cooled, and then refrigerated. The minimum gelation concentration was identified as the lowest concentration that remained solid when the tube was inverted.

### 2.8 Water holding capacity

The measurement of water retention ability was conducted by Foh et al. (2012). One gram of each protein isolate sample was mixed with 10 mL of distilled water for 30 s and then centrifuged at 2500×*g* for 25 min. The volume of the supernatant was then measured. Water holding capacity was expressed as mL of water absorbed per gram of protein sample.

### 2.9 Scanning electron microscopy

The protein samples were analyzed for their internal structure using an S-480 model scanning electron microscope (SEM) running at 15 kV. To prepare the samples for examination, they were coated with a layer of gold using an ion sputter coater (MCM-100, SEC) and examined at a magnification of 1000× (X 1.0 K) and 20 kV.

### 2.10 Sulfhydryl (R–SH) group content

The amount of active sulfhydryl groups of the isolated protein samples was determined using Ellman’s (1959) [18]. Protein samples (1 mg/mL) were dissolved in a Tris-glycine-EDTA-urea buffer. Ellman’s reagent (DTNB) was added, and the absorbance at 412 nm was measured after 25 min. The sulfhydryl content was calculated using a molar extinction coefficient of 13,600 M^−1^cm^−1^. Buffer alone served as a blank. The sulfhydryl content (µmol/g) was calculated using the formula: SH (µmol/g) = (A412 × 73.53) / protein concentration (mg/mL). Note: This buffer system primarily detects total sulfhydryl content. For quantifying reactive sulfhydryl groups, a reducing environment without urea may be more appropriate.

### 2.11 SDS-PAGE

Proteins were prepared in a homogeneous phase. Samples were first treated with loading buffer (Laemmli buffer containing SDS, Tris-HCl, glycerol, β-mercaptoethanol, and bromophenol blue), then incubated at 95 °C for 5 min. The SDS-PAGE was performed on a 12 % resolving gel and 5 % stacking gel, based on the method described by Harlow and Lane [13].

### 2.12 Particles size distribution

Particle size of the isolated proteins was measured using a Malvern Instruments laser particle size analyzer. Following Gao et al. (2022), samples (0.1 % w/v in pH-adjusted deionized water) were analyzed at 25 °C, with each measurement repeated three times.

### 2.13 Fourier transform infrared (FTIR) spectroscopy analysis

The secondary structure of the isolated proteins was determined by FTIR spectroscopy. Spectra were recorded at 25 °C with a resolution of 400–4000 cm^−1^. The proportions of secondary structure components were calculated from absorbance data between 1600 and 1700 cm^−1^. Characteristic peaks for β-sheet (1610–1640 cm^−1^ and 1670–1690 cm^−1^), α-helix (1650–1660 cm^−1^), random coil (1640–1650 cm^−1^), and β-turn (1660–1670 cm^−1^ and 1690–1700 cm^−1^) were identified, and their relative abundances were used to determine the overall secondary structure composition [20].

### 2.14 Sensory characteristics

Sensory evaluation of a 1 % (w/v) fish protein powder solution (1 g in 100 mL distilled water) was conducted according to Abdollahi & Undeland (2018). Solutions were prepared 1 h prior, chilled, and evaluated in duplicate by a trained sensory panel. Odor, taste, and aroma were scored on a 0–8 hedonic scale (Table 1). A 10-minute interval separated evaluations, with mouth rinsing between samples. Evaluation criteria included odor (fishy, pleasant), taste (intensity and balance), color (light to dark), and overall acceptability. Scores from 0 to 8 represented 'dislike extremely' to 'like extremely'.

### 2.15 Analysis of antioxidant activity

#### 2.15.1 DPPH radical scavenging activity

To evaluate the free radical scavenging activity against diphenyl picrylhydrazyl (DPPH), the method described by Shimada et al. [22] was employed with slight modifications. A 0.16 mM DPPH solution was prepared using 96 % ethanol. Subsequently, 500 µL of the sample at concentrations of 1, 0.75, and 0.5 mM was added to 500 µL of the DPPH solution and vortexed for 1 min. The optical absorbance of the samples was measured after 30 min of incubation at room temperature in the dark, at a wavelength of 517 nm, using a spectrophotometer (ELISA Reader Epock, USA) in triplicate. Ascorbic acid at a concentration of 0.1 mg/mL was used as the control for evaluation.

#### 2.15.2 ABTS radical cation scavenging activity

This test was conducted by the previously reported method by Alemán et al. [23]. For the preparation of the ABTS radical solution, 7 mM ABTS and 2.45 mM potassium persulfate were mixed to oxidize the ABTS, and the mixture was then kept at 25 °C in the dark for 16 h. After this period, the resulting solution was diluted with distilled water until an absorbance of 0.70 ± 0.02 was achieved at a wavelength of 734 nm. Subsequently, 20 µL of the sample was mixed with 980 µL of the diluted ABTS solution and incubated at 30 °C in the dark for 10 min. The absorbance of the samples was measured at a wavelength of 734 nm in triplicate using a spectrophotometer (ELISA Reader Epock, USA). Ascorbic acid was used as a comparison standard, and the percentage of ABTS radical inhibition was calculated using the following formula:

ABTS radical scavenging activity (%) = $\frac{A0-A}{A0}\times$100 %.

#### 2.15.3 Ferric reducing antioxidant power (FRAP)

To evaluate the iron-reducing activity, the method described by Oyaizu [24] was employed. Briefly, 100 µL of the sample was mixed with 250 µL of 0.2 M phosphate buffer (pH 6) and 250 µL of 1 % potassium ferricyanide. The resulting mixture was incubated at 50 °C in a water bath for 30 min. After incubation, 250 µL of 10 % trichloroacetic acid was added to the mixture. The solution was then centrifuged at 10,000 rpm for 10 min. Finally, 500 µL of the supernatant was mixed with 250 µL of distilled water and 50 µL of 0.1 % ferric chloride. After allowing color development for 10 min, the absorbance of the final solution was measured at a wavelength of 700 nm in triplicate using a spectrophotometer (ELISA Reader Epock, USA).

## 3 Results and discussion

### 3.1 Yield of protein extraction and solubility during pH-shift processing with and without ultrasound

**Fig. 1:**
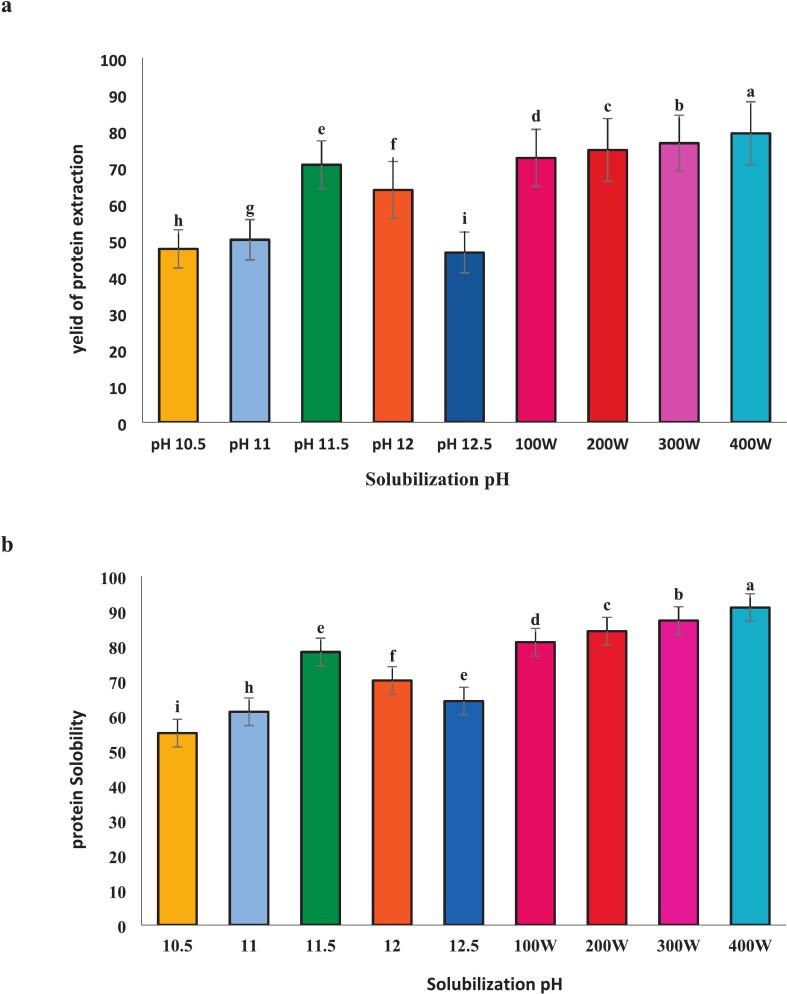
The impact of solubilization pH and ultrasonication power on recovered protein isolate and solubilization yield, isolated protein from the head of Acipenser baerii was investigated Alkaline pH shifting and then with an ultrasonic. Error bars represent the standard deviation (SD) of [10.5, 11, 11.5, 12, 12.5, 100,200,300 and 400 W] independent measurements. Distinct letters above the columns indicate a significant difference (P < 0.05).

(Fig. 1 a and b) shows the Yield of protein extraction and protein solubilization from *A. baerii* head using two methods: pH-shift alone and pH-shift combined with ultrasonication. The highest yield (78.29 %) was achieved at pH 11.5, consistent with findings in other species such as silver carp (*Hypophthalmichthys molitrix,* 79.80 %) [25] and tilapia (*Oreochromis niloticus,* 79.82 %) [17] under alkaline conditions. Minor discrepancy may be attributed to differences in fish species and centrifuge capacity, which significantly influence the extraction yield. The lower yields at higher pH values likely result from protein aggregation and increased size under highly alkaline conditions, reducing solubility [26]. As can be seen from the results, the highest protein solubility (73.70 %) was also observed at pH 11.5. This alkaline pH increases protein solubility due to enhanced charge repulsion between protein chains. However, solubility decreased at even higher pH values, likely due to protein aggregation [10,21]. This pattern aligns with findings from previous studies conducted on other fish species, such as Rainbow Trout (*Oncorhynchus mykiss*) [27]. Therefore, pH 11.5 was selected for further experiments combining pH-shift with ultrasound at 100, 200, 300, and 400 W. Fig. 1 shows that at pH 11.5, 400 W ultrasound yielded the highest protein yield (91.06 %) and solubility (91.52 %). It can be concluded that both the pH and the ultrasound power significantly affect protein extraction efficiency (P < 0.05). Compared to the treatment without ultrasound, both yield and solubility significantly increased with increasing ultrasound power (100–400 W). This can be attributed to the fact that ultrasound waves could dissolve the insoluble proteins in water, and reducing sediment trapping. Previous studies have also shown that high-intensity ultrasound markedly increased the solubility of isolated fish proteins [26,28].

### 3.2 Amino acid composition

According to (Table 1) at pH 11.5, the levels of essential amino acids such as histidine, valine, threonine, phenylalanine, leucine, isoleucine, and lysine increased compared to other alkaline treatments and the initial sample. This elevation may be attributed to the elimination of collagen impurities, such as bone and skin, which contain substantial amounts of non-essential amino acids, including glycine and proline. This finding aligns with research indicating that the levels of glutamic acid and aspartic acid also increased [29]. In the present study, all essential amino acids present in the isolated proteins were much higher than the recommended levels for adults, according to WHO/FDA/FAO/UNU (2007).

**Table 1:** Amino acid composition from the head of *A. baerii* in alkaline pH shift.

**Amino acid**							
	By-product	pH 10.5	pH 11	pH 11.5	pH 12	pH 12.5	FAO/WHO mg/g protein
Aspartic acid	12.26 ± 1.6	10.36 ± 1.4	8.93 ± 1	9.56 ± 1.3	8.33 ± 1	6.72 ± 1	
Glutamic acid	10.20 ± 1.8	7.00 ± 1.1	8.26 ± 1.2	9.60 ± 1.1	7.63 ± 1.4	9.03 ± 1.9	
Serin	12.82 ± 1.5	8.82 ± 0.8	9.31 ± 1.1	10.23 ± 1.2	7.44 ± 0.7	6.29 ± 0.8	
Histidine*	4.63 ± 0.7	7.52 ± 0.9	8.32 ± 0.7	10.90 ± 1.1	9.06 ± 0.9	6.12 ± 0.9	1.5 (3)
Glycine	7.43 ± 1.1	3.05 ± 1	6.02 ± 0.8	6.62 ± 0.6	5.36 ± 0.6	4.48 ± 0.7	
Threonine*	1.16 ± 0.2	3.24 ± 0.5	5.92 ± 0.7	6.86 ± 0.8	5.04 ± 0.6	4.12 ± 0.4	2.3 (3.1)
Arginine	8.98 ± 0.9	4.39 ± 0.6	5.31 ± 0.9	7.86 ± 0.9	6.10 ± 0.6	6.12 ± 1	
Alanine	3.52 ± 0.4	7.03 ± 1	6.32 ± 0.8	7.96 ± 0.9	4.38 ± 0.7	5.40 ± 0.6	
Tyrosine	1.2 ± 0.3	3.9 ± 0.5	3.01 ± 0.6	2.22 ± 0.4	4.85 ± 0.8	3.41 ± 0.5	
Methionine*	6.43 ± 0.8	4.22 ± 0.6	5.06 ± 0.6	6.97 ± 1	6.11 ± 0.8	5.73 ± 0.8	1.6 (4.2)
Valine*	4.52 ± 0.6	8.02 ± 1.1	7.3 ± 0.9	12.83 ± 2	10.80 ± 1	9.35 ± 1.4	3.9 (5.5)
Phenylalanine*	1.44 ± 0.2	2.10 ± 0.3	1.83 ± 0.2	4.86 ± 0.8	3.01 ± 0.6	2.73 ± 0.4	1.9 (7.2)
Isoleucine*	4.92 ± 0.6	6.24 ± 0.9	5.73 ± 0.5	7.79 ± 0.9	8.82 ± 1.1	7.02 ± 0.7	3 (3.2)
Leucine*	5.5 ± 0.9	8.12 ± 0.9	13.21 ± 1.4	16.63 ± 2.4	9.56 ± 1.3	7.63 ± 1.1	5.9 (6.6)
Lysine*	2.54 ± 0.3	3.75 ± 0.5	4.23 ± 0.5	8.16 ± 1.1	3.06 ± 0.5	1.03 ± 0.1	4.5 (5.7)
EAA	30.00	39.99	45.71	68.16	50.44	39.62	
NEAA	87.59	87.82	98.82	129.10	99.60	85.23	
EAA/AA	0.34	0.45	0.46	0.52	0.50	0.46	

EAA: Total essential amino acid, NEAA: Total non-essential amino acid, AA: Total amino acid.

*Shows essential amino acids.

### 3.3 Foaming capacity and stability

**Fig. 2:**
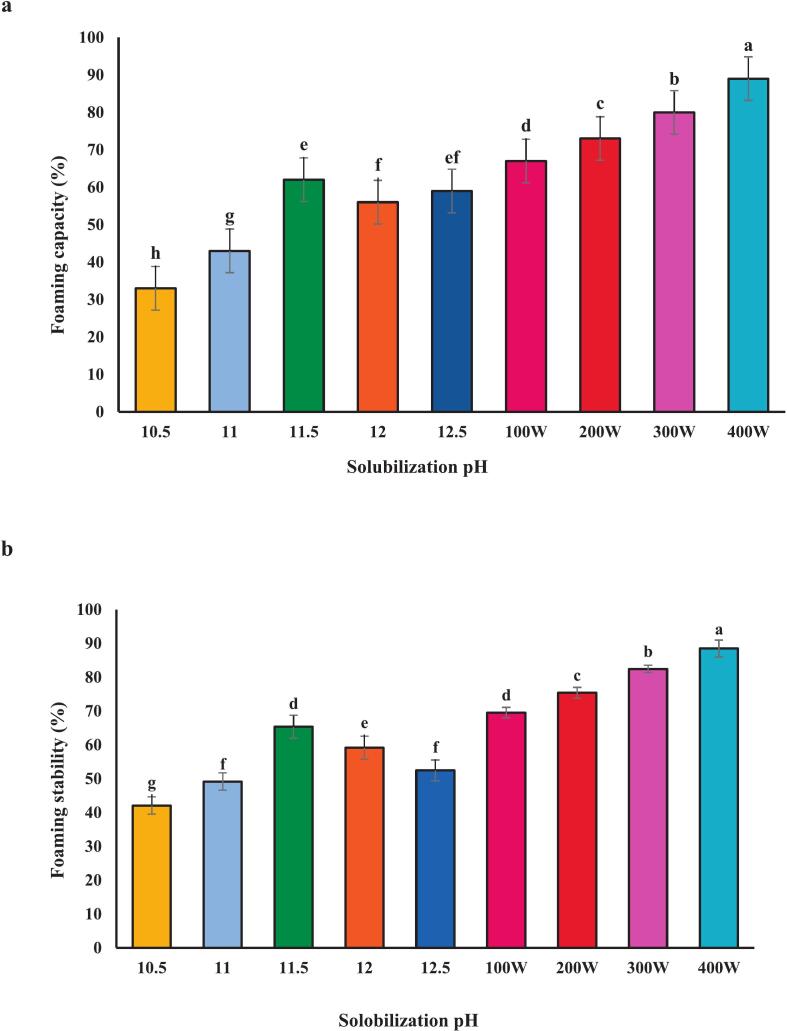

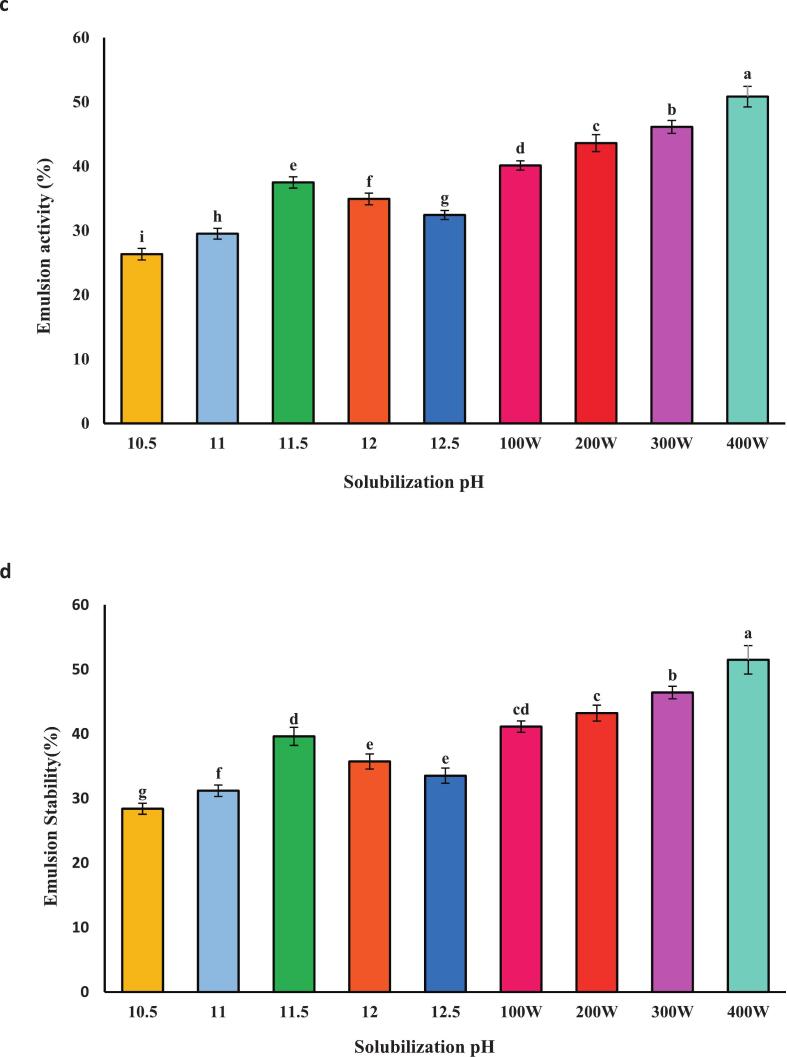

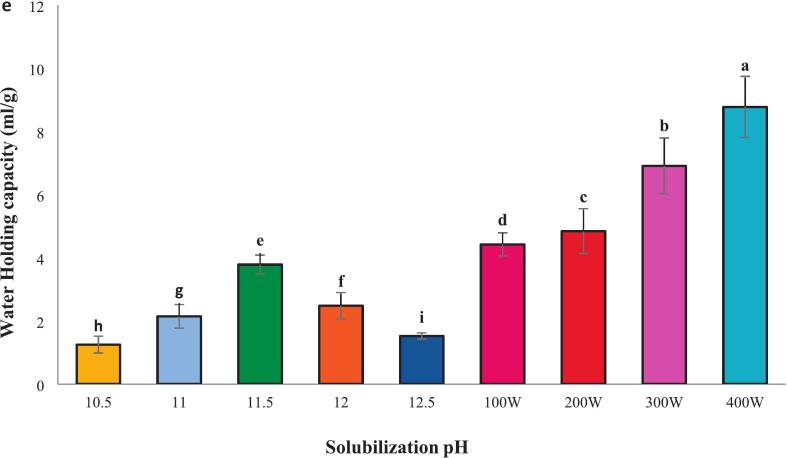
Functional properties include**:** Foaming capacity **(a)** foam stability **(b)**, Emulsion activity **(c)** Emulsion stability **(d)** and Water holding capacity **(e)** of proteins recovered alkaline pH change processing and combination of selected pH 11.5 with ultrasound waves of different powers.. Error bars represent the standard deviation (SD) of [10.5, 11, 11.5, 12, 12.5, 100,200,300 and 400 W] independent measurements. Distinct letters above the columns indicate a significant difference (P < 0.05).

The foaming capacity of proteins is crucial in food applications. (Fig. 2 a and b) show that isolated proteins exhibited increased foaming capacity and stability at pH 11.5. This enhancement is attributed to the improved dispersion of proteins at the air–water interface due to increased net charge under alkaline conditions, facilitating greater foam formation [30]. Gao et al. [19] reported a similar alkaline extraction-induced increase in foaming capacity, supporting our findings of 65.35 % at pH 11.5 (Fig. 2a). Foam stability is also highly influenced by pH, with the highest value of 62.00 % observed at pH 11.5 in Fig. 2b. Further enhancement was observed when combining pH 11.5 with 400 W of ultrasound (Fig. 2 a and b): foaming capacity reached 89.00 %, and stability reached 88.46 %. This synergy suggests that ultrasound, in addition to alkaline pH, significantly improves foam formation and stability (*P* < 0.05), assuming statistical significance was determined). The mechanism likely involves ultrasound-induced protein denaturation, reduced particle size, and increased surface area [31]. These findings align with previous research conducted by Jambrak et al. [32], who reported an enhancement in the foaming capacity and stability of cheese whey protein after ultrasound treatment. Altogether, both pH and ultrasound significantly impact the foaming capacity and stability of the isolated proteins, with the combination of alkaline pH and ultrasound yielding optimal results.

### 3.4 Emulsion activity and stability

**Fig. 3:**
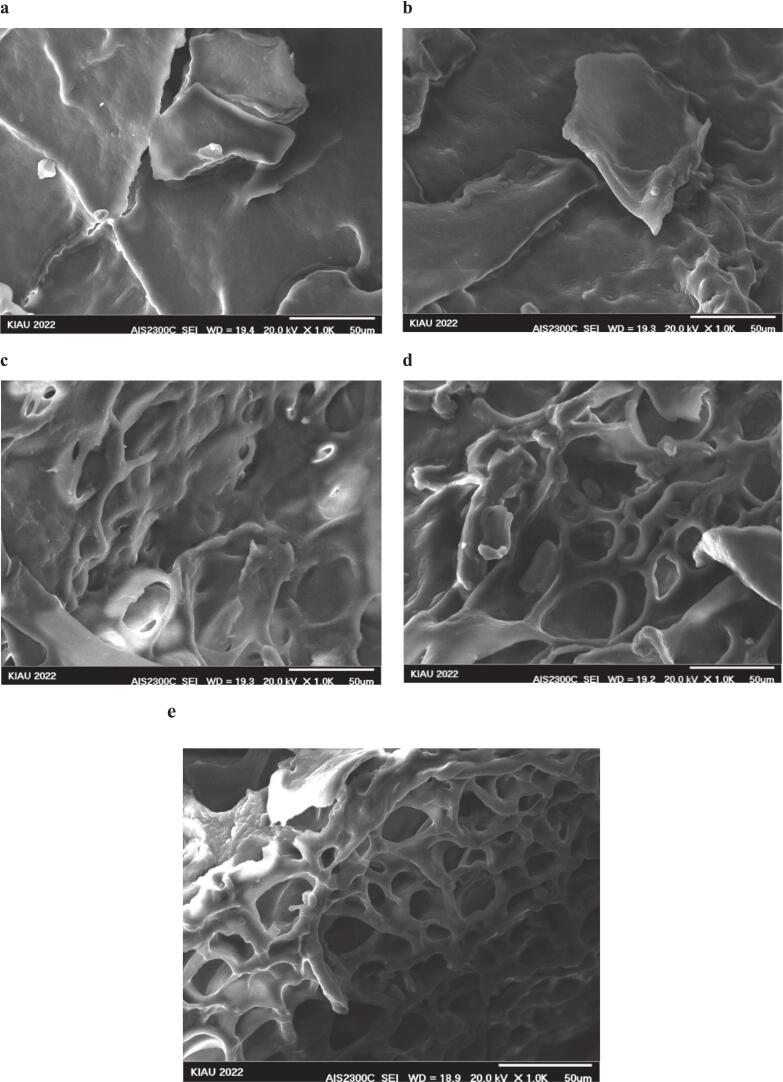
SEM images of isolated fish proteins under different treatments: a (pH 5.11), b (100 W), c (200 W), d (300 W), and e (400 W), captured at a magnification of 1000× (X 1.0 K).

The emulsification capacity of isolated protein from *A. baerii* was pH-dependent reflecting the influence of pH on the balance between the protein's hydrophobic and hydrophilic properties. Proteins possess both hydrophilic (water-loving) and hydrophobic (water-repelling) regions, impacting their emulsifying ability. As depicted in (Fig. 2 c and d), the highest emulsification capacity (37.48 %) and the emulsion stability (38.62 %) was achieved at pH 11.5. This is consistent with findings on tilapia muscle proteins [33], likely due to the exposure of hydrophobic groups upon protein denaturation. Fig. 3b demonstrates that combining pH 11.5 with 400 W of ultrasound further enhanced emulsification capacity (82.50 %) and stability (48.50 %). Ultrasound treatment increases protein surface area and alters the protein structure, promoting interfacial adsorption. A portion of the protein actively contributes to the formation of the surface layer, enhancing emulsification efficiency. Moreover, increased surface hydrophobicity and increased molecular flexibility during protein denaturation lead to more effective absorption of protein molecules at the oil–water interface [10]. Proteins treated with ultrasound typically exhibit increased surface hydrophobicity, intensifying emulsifying activity by interacting with neighboring molecules at the interface. Partial denaturation of proteins induced by ultrasound waves, along with structural disruption and changes in protein particle size distribution, can enhance the ability to absorb at the oil–water interface [31]. The results indicate that emulsification capacity and stability in protein samples prepared with 300 W (68.58 % and 90.87 %, respectively) were higher than those prepared at 100 W (68.40 % and 74.72 %) [31].

### 3.5 Gel formation

The gel formation of proteins is depending on their partial denaturation [34]. Table 2 indicates that the lowest gel formation was occurred at pH 11.5. Under alkaline pH conditions, the net charge of proteins and the electrostatic repulsion between myofibrillar proteins increased, which enhanced water retention within the actomyosin structure, ultimately affecting gel strength. These findings are consistent with those of other studies [35,36]. According to the results regarding the combined effect of pH and ultrasound on gel formation concentration presented in Table 2, increasing the ultrasound power to 400 W resulted in greater gel strength. This improvement could be attributed to ultrasound waves reducing the size of protein particles and homogenizing them, thereby improving gel strength. These findings align with those of Li et al. [37], who compared gels exposed to ultrasound with those that were not and found that ultrasound significantly increased gel strength in by-product samples.

**Table 2:** Gel formation of proteins isolated using the classic pH-shift processing and pH 11.5 combination with ultrasonication at various solubilization pH values and ultrasound.

**pH Solubilization**	**Gel formation**
10.5	20 ± 1 ^a^
11	20 ± 2 ^a^
11.5	15 ± 2/64^b^
12	20 ± 3 ^a^
12.5	20 ± 1 ^a^
100 W	15 ± 2^b^
200 W	15 ± 2/64^b^
300 W	15 ± 3^b^
400 W	10 ± 3^c^
Distinct letters showed a significant difference (P < 0.05).	

### 3.6 Water holding capacity

As shown in (Fig. 2 e), the highest water-holding capacity was observed at pH 11.5 (3.78), which can be attributed to changes occurring in the protein structure and charged groups. This results in the exposure of water-binding regions and an increased ability for water to bind with the protein as its polarity increases [26]. The highest water-holding capacity at pH 11.5, combined with ultrasonic treatment at 400 W (8.77), is associated with the smaller particle size of the protein and the alkaline pH. Additionally, the protein’s surface becomes more likely to interact with water due to exposure to ultrasonic waves, leading to enhanced water retention in the protein sample. Similar findings were reported by Zhang et al. [38], who noted an increase in water-holding capacity in myofibrillar protein with the increase of ultrasonic power up to 600 W and suggested that this could be due to the tight entrapment of water molecules in the small pores of the homogeneous protein structure.

### 3.7 Scanning electron microscopy (SEM)

(Fig. 3) Illustrates the fine structure of isolated protein at pH 11.5 in combination with ultrasound treatment. The results indicate that increasing ultrasound power in isolated fish protein leads to the formation of cavities on the surface of protein sheets. When exposed to ultrasound waves at 400 W, the isolated protein shows that higher ultrasound power results in the formation of smaller protein particles. The network-like structure observed in SEM images may be attributed to partial protein aggregation during sample drying or preparation, resulting in a continuous structure. This phenomenon can be attributed to the explosion caused by ultrasonic cavitation, which facilitates the entry of desired components into the solvent and enhances protein extraction efficiency at 400 W compared to other treatments (Pezeshk et al., 2021). This finding is similar to the result obtained in the study conducted by Chen et al. (2019). Investigations into the morphological structure of isolated protein from rainbow trout (*Oncorhynchus mykiss*) waste concluded that higher ultrasound power can lead to the production of smaller protein particles, which aligns with the results obtained by Pezeshk et al. (2021).

### 3.8 Sulfhydryl group content

**Fig. 4:**
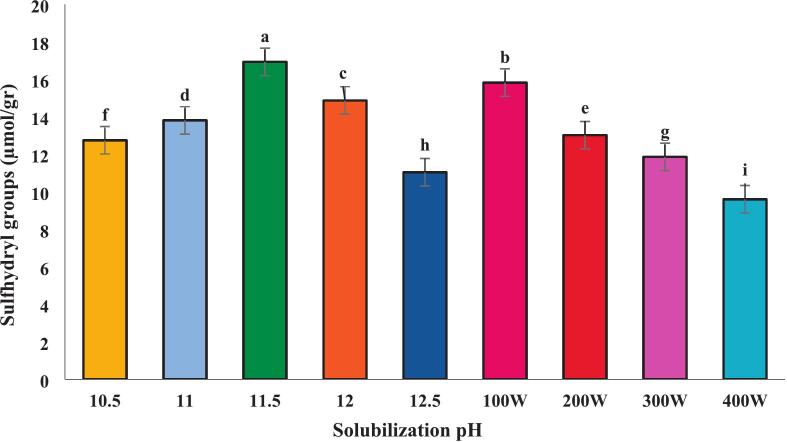
Reactive sulfhydryl groups of proteins recovered alkaline pH change processing and combination of selected pH 11.5 with ultrasound waves of different powers.. Error bars represent the standard deviation (SD) of [10.5, 11, 11.5, 12, 12.5, 100,200,300 and 400 W] independent measurements. Distinct letters above the columns indicate a significant difference (P < 0.05).

The quantity of reactive sulfhydryl groups in proteins obtained through traditional pH shift processing and/or in combination with ultra-sonication at pH 11,5, across different power levels, is shown in (Fig. 4) The free sulfhydryl groups in protein samples at alkaline pH levels were measured, revealing that at pH 11.5, the highest concentration was 16.60 µmol/g. Jiang et al. (2009) suggested that this increase could be attributed to the higher solubility of this protein under these conditions. Conversely, the isolated protein at pH 11.5 combined with ultrasound at 400 W exhibited the lowest concentration of 9.60 µmol/g. This reduction may be due to the formation of disulfide bonds induced by the intense ultrasound waves [39].

### 3.9 Distribution of molecular weight

**Fig. 5:**
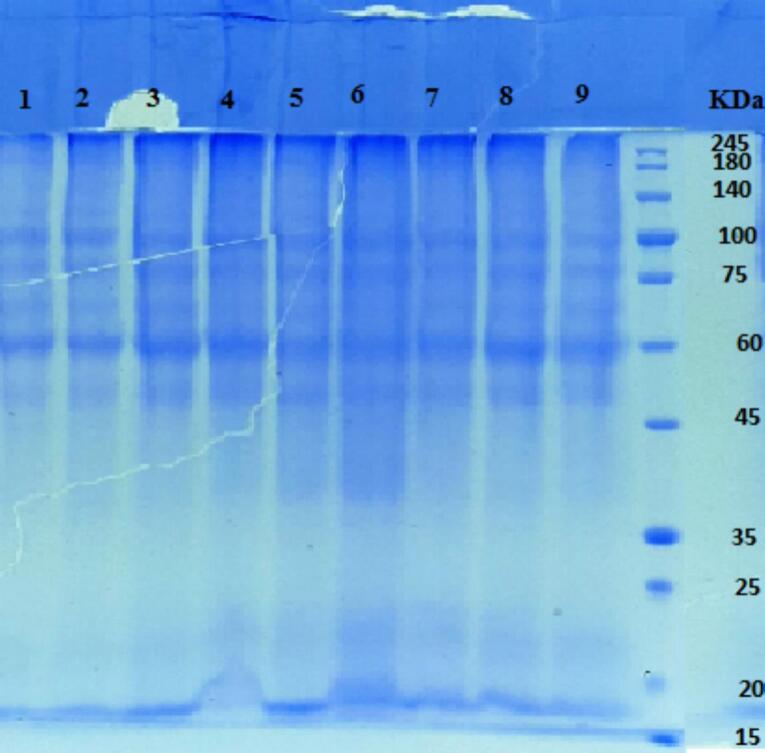
SDS-PAGE gel of proteins isolated from the head of *A. baerii* using the alkaline pH-shift (10.5, 11, 11.5, 12, and 12.5) processing and pH 11.5 combination with ultra-sonication at various power (100, 200, 300, and 400 W) for 15 min.**1:** pH 12.5, **2:** pH 12, **3:** pH 10.5, **4:** pH 11, 5: pH 11.5, **6:** pH 11.5 + 400 W, **7:** pH 11.5 + 300 W, **8:** pH 11.5 + 200 W, **9:** pH 11.5 + 100 W.

The arrangement of polypeptides in proteins at alkaline pH and their combination with ultrasound is illustrated in (Fig. 5) Initially, in the protein samples at alkaline pH, an increase in gel color was observed as the molecular weight decreased from 245 kDa to 45 kDa at pH 11.5 (band number 3). This indicates a higher amount of extracted protein in this treatment. Furthermore, no decrease in gel color was observed in the range from 45 to 15 kDa, suggesting that there is no significant amount of isolated protein with low molecular weight in this yield. In bands 6 to 9 in the figure, where protein samples at pH 11.5 were subjected to ultrasound at powers of 100, 200, 300, and 400 W, the intensity of gel color in band 6 compared to the control band (without ultrasound) at pH 11.5 became more pronounced, indicating a dependency on the amount of extracted protein. The molecular weight ranged from 245 kDa to 60 kDa across the other ultrasound-combined treatments. Notably, at 400 W, the highest amount of protein was displayed in the gel, ranging from 45 kDa to 15 kDa, with an increase in gel color observed in band 6. As ultrasound power decreased, the gel color also diminished accordingly. This phenomenon can be attributed to the impact of ultrasound intensity on the protein molecule’s structure, resulting in a decrease in its molecular weight [40].

### 3.10 Particle size in protein solution

**Fig. 6:**
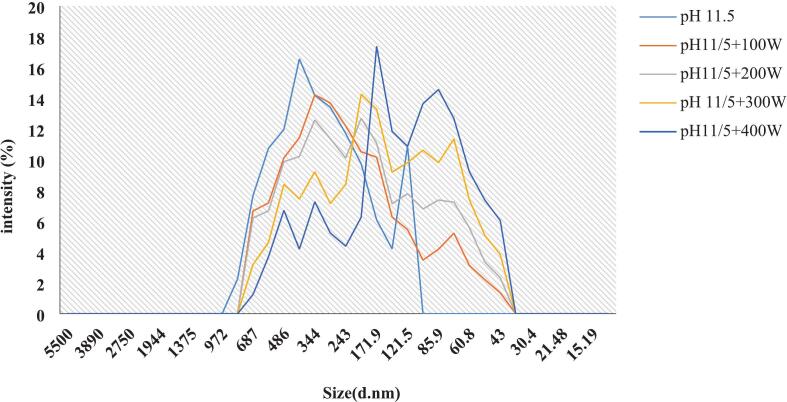
Particle size distribution FPI in alkaline pH 11.5 and combined with ultrasound at different power.

The size of proteins in a liquid solution impacts their functional characteristics, including their ability to foam, dissolve, and form emulsions [41]. The effect of ultrasonic waves on the dimensions of isolated proteins from the head of *A. baerii* at a pH of 11.5 is illustrated in (Fig. 6) Increasing the ultrasound power to 400 W resulted in smaller protein particles compared to the control sample without ultrasound. This phenomenon is thought to be caused by the intense energy waves, cavitation, and turbulence produced by the ultrasound, which disrupt the non-covalent bonds between proteins, such as hydrogen bonding, electrostatic interactions, and hydrophobic interactions, resulting in a decrease in the size of protein particles [42,43]. These results align with earlier research that has noted the influence of ultrasound treatment on the size of particles in rapeseed proteins and soy proteins [44].

### 3.11 Secondary structure

**Fig. 7:**
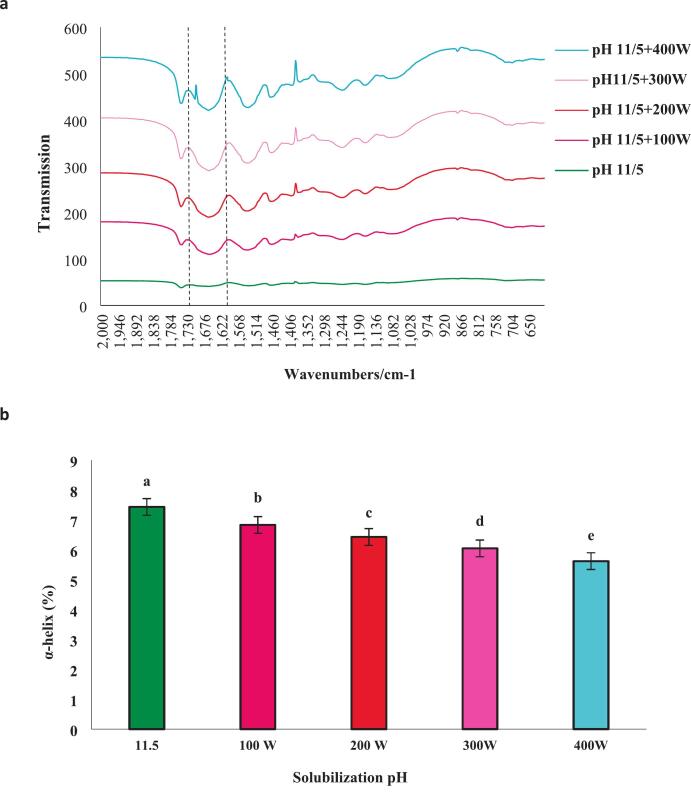

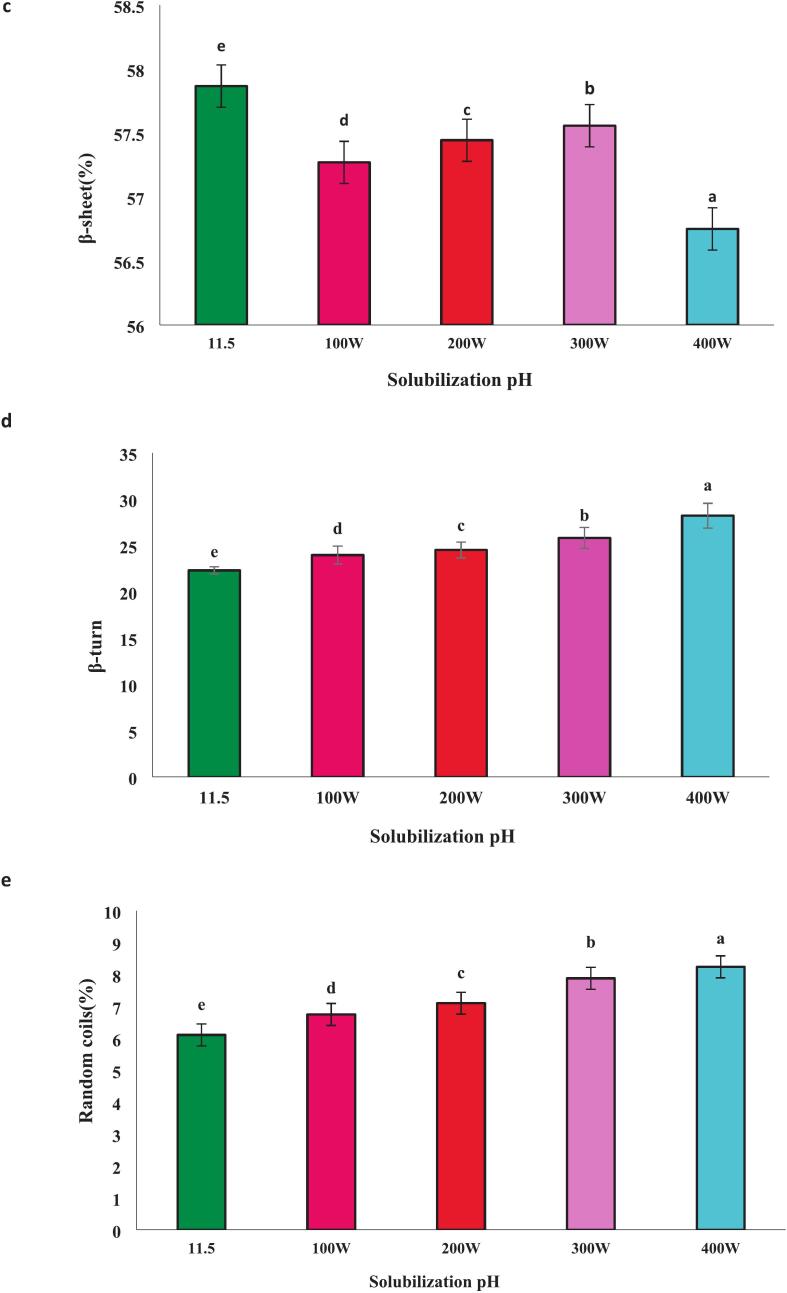
The FTIR spectra of proteins extracted from the head of *A. baerii* through the conventional pH 11.5 process and its amalgamation with ultrasonication are (100,200,300 and 400 W) for 15 min. α-helix **(b)**, β-sheet **(c)**, β-turn **(d)** and random coils **(e)**.. Error bars represent the standard deviation (SD) of [10.5, 11, 11.5, 12, 12.5, 100,200,300 and 400 W] independent measurements. Distinct letters atop the columns signify significant differences (P < 0.05).

FTIR spectroscopy was employed to analyze the secondary structural components of proteins, such as α-helix, β-sheet, β-turn, and random coil, to provide a rationale for the variations observed in their foaming and emulsifying properties. As shown in (Fig. 7a(the secondary structural components of isolated protein samples at pH 11.5, in combination with ultrasound at power levels of 100, 200, 300, and 400 W, are presented. The amide I band, characterized by a peak in the range of 1600–1700 cm^−1^, corresponds to the stretching and vibration of the C=O group in the structure. Overall, FTIR spectroscopy was utilized to identify the secondary structural components of proteins, aiming to elucidate the observed distinctions in their emulsifying properties and foam formation. Isolated proteins from *A. baerii* at pH 11.5, when combined with ultrasound at 400 W, significantly reduced the α-helix content compared to the treatment without ultrasound. Fig. 7b illustrates that ultrasound caused a modification in protein structure, given that α-helices play a crucial role in maintaining protein integrity [45].

The secondary structure of proteins is contingent upon the amino acid sequence and the interactions among various segments of the molecule. Ultrasound has the potential to disrupt hydrogen bonds within proteins and hydrophobic interactions within α-helices, potentially leading to protein unfolding, which affects their structure and enhances molecular flexibility [46]. Research investigating the role of secondary structures in myosin at various pH levels concluded that as pH increased from 5.5 to 7, β-sheets decreased from 41.2 % to 5.1 %. Furthermore, the α-helix content gradually decreased from 87.7 % to 36 % as pH increased [47]. Based on Fig. 7c, d, and e (associated with β-sheets, β-turns, and random coils, respectively), proteins exposed to ultrasound exhibited a notable enhancement in the analyzed parameters compared to isolated proteins without ultrasound treatment (P < 0.05). These findings suggest that ultrasound has the potential to selectively disrupt particular types of hydrogen bonds, resulting in alterations to certain α-helical, β-sheet, β-turn, and random coil structures. Thus, the combination of ultrasound with the alkaline process has led to the weakening the rigid protein structures, enhancing their flexibility, and causing changes in their secondary structure. Additionally, it was observed that ultrasound results in a reduction in the α-helical component and an increase in the content of β-sheets [48].

### 3.12 Sensorial properties

**Fig. 8:**
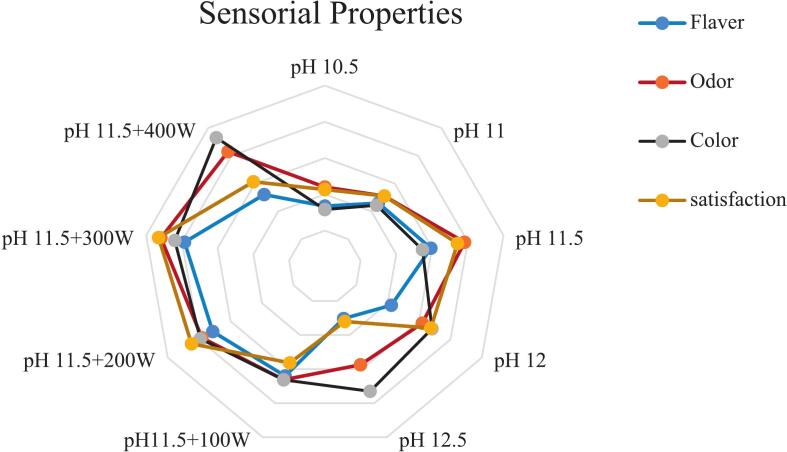
Sensorial attributes (flavor, odor, Color, and satisfaction) of protein isolate from the head of *A. baerii* with pH shift method and combination with ultrasound in various power.

As shown in (Fig. 8) sensory properties including odor, color, and flavor were evaluated at alkaline pH levels. At pH 11.5, and particularly in combination with ultrasonic waves at a power of 400 W, the highest levels of satisfaction regarding odor and flavor were observed. This enhancement can be linked to the increased levels of glutamic acid and aspartic acid (Table 1), which contribute to more favorable functional properties. The increase in these two amino acids in the protein sample has resulted in a more desirable odor and flavor. Furthermore, the sample treated with ultrasound at 400 W exhibited a darker color compared to the other treatments, likely due to the presence of pigments such as hemoglobin in the related product [49].

### 3.13 Antioxidant activities

**Fig. 9:**
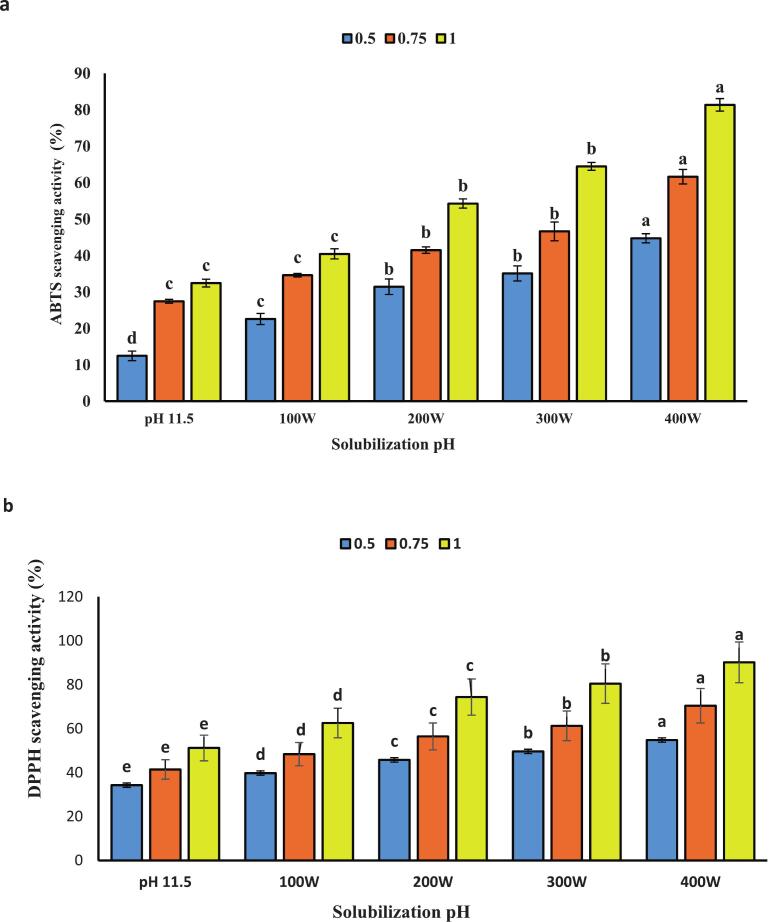

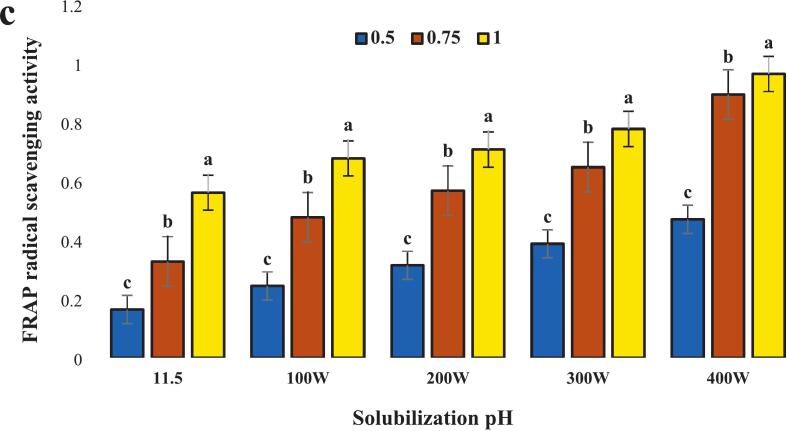
Antioxidant activity included: DPPH radical scavenging activity **(a)** ABTS radical scavenging activity and **(b)** ferric reducing antioxidant power (FRAP) **(c)** protein isolated from the head of *A. baerii* using the classic pH 11.5 process and its amalgamation with various ultrasonication.. Error bars represent the standard deviation (SD) of [10.5, 11, 11.5, 12, 12.5, 100,200,300 and 400 W] independent measurements. Distinct letters atop the columns signify significant differences (P < 0.05).

The radical scavenging activity of the isolated protein was assessed using ABTS and DPPH free radicals at pH 11.5, in combination with ultrasound waves at 400 W, as illustrated in (Fig. 9 a and b) where the highest levels were observed. Generally, this increase was concentration-dependent, showing a direct correlation with the percentage of inhibition. This can be explained by the fact that antioxidant capacity is primarily determined by peptides with low molecular weight. Ultrasonic cavitation facilitates the release of these low molecular weight peptides, enhancing their likelihood of interaction with free radicals [50].

One study investigated the effect of ultrasound on the antioxidant properties of lupin protein and concluded that the power of ultrasound enhances these properties. The authors suggested that the increase in antioxidant capacity could be attributed to the protein’s exposure to ultrasound waves, which reveals previously hidden amino acid residues and side chains possessing antioxidant capacity. These residues are commonly concealed within the three-dimensional structure of protein molecules [11,51].

In (Fig. 9c) the iron-reducing power (FRAP) of the isolated protein was measured at pH 11.5 in combination with ultrasound waves. The results indicated that it exhibited the highest FRAP value was observed at 400 W, and this increase was concentration-dependent. This suggests that ultrasound power induces changes in the protein composition, leading to the release of peptides capable of interacting with iron ions and donating electrons to them. Ultrasound waves enhance hydrophobic sites, resulting in an increased in electron-donating capability and iron-reducing power [52].

## 4 Conclusion

Siberian sturgeon (*A. baerii*) is one of the most economically significant cultivated fish species worldwide. The isolated protein from the fish head was produced using an alkaline pH adjustment method, with pH levels of 10.5, 11, 11.5, 12, and 12.5. The extracted protein was assessed, and its yield, solubility, and nutritional value, focusing on its essential amino acid content at pH 11.5. At this pH, the isolated protein underwent ultrasound treatment at various power levels (100, 200, 300, and 400 W) and analyzed for structural, functional, sensory, and antioxidant properties. The findings indicated that at pH 11.5, coupled with ultrasound at 400 W, functional properties such as foaming capacity, emulsification, gel formation, and water-holding capacity exhibited superiority compared to other treatments (p < 0.05). Structural analysis revealed that increases in ultrasound power led to the generation of smaller protein particles and the formation of voids on the protein surface. The free sulfhydryl (SH) group showed the lowest amount with increasing ultrasound power (p < 0.05). In molecular weight analysis, the fractures created in the protein structure due to increased ultrasound power resulted in the lowest molecular weight being observed at 400 W. Results from Fourier-transform infrared spectroscopy (FTIR) demonstrated that an increase in ultrasound power to 400 W altered the protein structure, leading to a reduction of alpha-helix content (p < 0.05). Sensory evaluation revealed that the combined treatment at pH 11.5 and 400 W received the highest preference among the assessors. Additionally, antioxidant property assessments indicated a significant increase in the inhibition of DPPH and ABTS radicals, as well as iron-reducing capacity, with the of isolated protein at 400 W.

## CRediT authorship contribution statement

**Seyedeh Mona Hosseini Choupani:** Writing – review & editing, Writing – original draft, Validation, Software, Methodology, Investigation, Formal analysis, Data curation, Conceptualization. **Masoud Rezaei:** Writing – review & editing, Validation, Supervision, Investigation, Funding acquisition, Conceptualization. **Samaneh Pezeshk:** Writing – original draft, Validation, Methodology, Investigation, Data curation, Conceptualization. **Shahab Naghdi:** Writing – review & editing, Writing – original draft, Data curation, Conceptualization. **Reza Tahergorabi:** Writing – review & editing, Funding acquisition.

## Declaration of competing interest

The authors declare that they have no known competing financial interests or personal relationships that could have appeared to influence the work reported in this paper.
